# To be objective in Experimental Phenomenology: a Psychophysics application

**DOI:** 10.1186/s40064-016-3418-4

**Published:** 2016-10-05

**Authors:** Roberto Burro

**Affiliations:** Department of Human Sciences, University of Verona, Lungadige Porta Vittoria, 17, 37129 Verona, Italy

**Keywords:** Experimental Phenomenology, Psychophysics, Rasch models, Conjoint measurement, Psychological objective method

## Abstract

**Background:**

Several scientific psychologists consider the approach for the study of perceptive problems of the Experimental Phenomenology is problematic, namely that the phenomenological demonstrations are subjectively based and they do not produce quantifiable results.

**Aim:**

The aim of this study is to show that Experimental Phenomenology can lead to conclusions objective and quantifiable and propose a procedure allowing to obtain objective measuring using the Rasch mathematical model able to describe the experimental data gathered in Experimental Phenomenology procedures.

**Method:**

In order to demonstrate this, a Psychophysics simulated study is proposed.

**Results/conclusions:**

It is possible to carry out a fundamental measurement starting from Experimental Phenomenology by way of the Theory of Conjoint Measurement.

## Introduction

### Background

Phenomenological analysis (Giorgi 1985) applies methods of phenomenological philosophy to psychological issues in order to find the meaning of direct experience, so that it is the experience itself that tells the true story.

Originally, phenomenology meant the attempt to obtain the things themselves, without being misled by prejudices (Valori 1967). In the field of Psychology, the most eloquent definition of phenomenology probably comes from Metzger (1941): “… to take the phenomenon simply as it is given, even if it appears unusual, unexpected, illogical, absurd, or contrary to unquestioned assumptions and familiar trains of thought. Let the things speak for themselves, without side glances at the well-known, at what has been learned earlier, at claims of logic, linguistic biases, and deficits of the vocabulary. Face the phenomenon with respect and sympathy, but question and distrust the presuppositions and conceptions with which the phenomenon in question has hitherto been grasped”. The natural science of perception, that is, Experimental Phenomenology, is carried out on this premise, and in accordance with that which Bozzi (2002) states, and by adopting the methods of scientific research that are so important to the natural sciences, by multiplying the observations and perfecting them in precise experimental designs, and by applying them to observable situations in controlled conditions and tracking down the logic itineraries that act as a go-between between the laboratory and common observed reality. After all, research such as that by Rubin, Wertheimer, Michotte, Johansson, Kanizsa and many other illustrious phenomenologists diverge as to methods, procedures and reasoning of the work of the ethologist just for the type of material observed alone: colour, transparency, geometry, etc., on the one hand and on the other hand that of the animal world. The perceptive event, which is the object of Experimental Phenomenology, exists only in as much as it is under observation and that observation takes place in the moment of the presence of the object (Stern 1897).

Experimental Phenomenology first obtains a description of a specific type of experience, starting from the responses given by a sample of subjects, and then educes differences and similarities in that experience, by extracting structural invariants (Spiegelberg 1972; Valle and Halling 1989). Kanizsa (1984) calls attention to the fact that Experimental Phenomenology does not limit itself to an inventory of phenomena, as is often believed. Its aim is much more ambitious: it is to find and analyse causal connections among observed phenomena, to individuate conditions that determine, foster and impede their appearance and their grade of evidence. All of this occurs without leaving the domain of phenomena, without resorting to neurophysiological and physical processes.

It is necessary to emphasize that a certain philosophical/scientific conviction, namely those who sustain that since immediate experience of perceived reality is continually evolving, therefore ungraspable, and not open to rigorous study, it has no reason for existing. Within this context, Palmer (2003), in a discussion of methodological approaches for the study of perceptive problems, says that phenomenological demonstration is a useful, but relatively blunt instrument for studying perceptual organization. There are two problems then for phenomenological demonstrations: they are subjectively based and they do not produce quantifiable results.

### Aim

The aim of this study is to show that Experimental Phenomenology can lead to conclusions objective and quantifiable. Regarding the first aforementioned point of Palmer, one can say that this scepticism derives from a sometimes incorrect distinction between the phenomenal terms, subjective as phenomenal is confused with subjective as idiosyncratic (Kubovy and Gepshtein 2003). The former means a characteristic of or belonging to reality as perceived before the intervention of consciousness; while the latter means something peculiar of a particular individual, arising from conditions within the brain or sense organs and not directly caused by external stimuli. In certain cases, subjective is prevalently phenomenal (the experience of the colour red is phenomenal, not idiosyncratic), while in other cases it is both things (the experience of beauty is phenomenal and idiosyncratic at the same time). Carrying out Experimental Phenomenology means studying the phenomenal, and not the idiosyncratic: in the opposite case it is simply the subjective experience that is studied.

With the first problem Palmer called attention to out of the way, the second problem that still needs discussing regards the quantification of results that can only be realized using a mathematical model able to describe the experimental data gathered (Kubovy 2002). The formal properties of a mathematical model in use must discriminate between quantification for evaluation (not rigorous method) and quantification for measurement (rigorous method). The deduction to be made is that a phenomenological description, in order to be considered scientifically authoritative, should rely on a measurement model of the results that leads to an scientific result.

The goal of the next sections is to propose a procedure that leads to rigorous measurements in places of research typical to phenomenological investigations, and to offer up a new point of view for psychological research and study.

### The relationship between numbers and measurements

It can be safely affirmed that all measurements are numbers, but not all numbers are measurements. Measuring procedures permit the use of numbers with the aim of reflecting on characteristics present at an empirical level. For a fuller discussion, see Krantz et al. (1971). Scientific evaluation needs genuine measurements and not just simple numerical labels, counts, raw scores and ranks. When numbers are used to describe results of an experiment, arithmetic is carried out: addition and division are used to calculate an average; subtraction is used for comparing and evaluating any improvements in performance; relationships are calculated to estimate possible benefits due to a treatment, etc. However, correct mathematical operations made ​​on numbers used improperly, apart from making very little sense, can also bring about misleading conclusions.

Unfortunately, it is not easy to have numbers that are measurements in the Psycho-Social Sciences, therefore also in Experimental Phenomenology. When an experimenter uses dichotomous scales of responses, categories such as: very much agree–agree–uncertain–disagree–strongly disagree; or category ratings in general (Torgerson 1958), it is presupposed that: the scale is suitable for obtaining the construct under question, the experimental subject is able to make evaluations that are constant over time, scale categories are accurately recorded, the distance between categories is constant, having a scores is like having a measurement. These are all suppositions.

However, starting from concrete dichotomous or ordinal observations whose raw scores have been obtained from categorical evaluations, good approximations of abstract measurements are possible. In other words, is possible to attribute at a number the status of measurement: with this perspective, characteristic procedures and concepts of Fundamental measurements are included (Campbell 1920; Luce et al. 1990). To simplify somewhat the complexity of the discussion, it can say that an objective measurement can be made if the gathered empirical data are described by a mathematical model that has three fundamental properties: linearity, stochastic independence, and specific objectivity. It is possible to sum up these three properties in one term with the so-called property of concatenation: if you add (or concatenate) to object mass 1 (or concatenate) a second object mass 2, a third object is obtained whose mass is equal to the first two added together. As banal as it may seem to a physicist, this proposition is elusive to the psychologist. How do you concatenate two motivations, two types of intelligences, two perceptive abilities, two emotional states, etc.?

However, the fundamental measurement is possible even without the property of concatenation: this is where conjoint measurement comes in (Luce and Tukey 1964).

The next section explains how obtain “fundamental measurement” in the framework of Experimental Phenomenology.

## Methods

### Formalisations

**Fig. 1:**
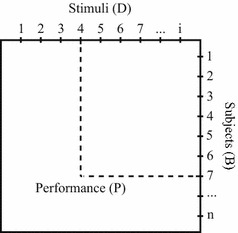
Two-dimensional graph of a stimulus/subject relationship

The theory of conjoint measurement says that a performance (P) is due to the conjoint effect of two parameters: a characteristic of a subject (B) and a characteristic of a stimulus (D). These parameters can be placed in a two-dimensional structure, as in Fig. 1.

It follows that every performance is a multiplicative transformation of B and D, that is

$$ P_{ni} = \frac{{B_{n} }}{{D_{i} }} $$

Using a logarithmic conversion the above formula can be converted into a type of linear transformation

$$ { \ln }({\text{P}}_{\text{ni}} ) = {\text{ln(B}}_{\text{n}} )- {\text{ln(D}}_{\text{i}} ) $$

It is possible to proceed with the replacement*s*

$$ {\text{ln(P}}_{\text{ni}} )=\uplambda_{\text{ni}} ;\quad {\text{ln(B}}_{\text{n}} )=\upbeta_{\text{n}} ;\quad { \ln }({\text{D}}_{\text{i}} ) =\updelta_{\text{i}} $$

Another simplification can be made

$$ \uplambda_{\text{ni}} =\upbeta_{\text{n}} -\updelta_{\text{i}} $$

**Fig. 2:**
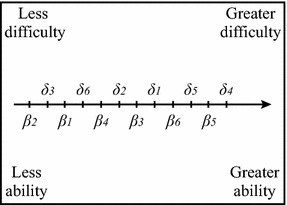
Hypothetical relationship between discriminative ability of subjects and difficulty of the stimuli to be discriminated

*β*_*n*_ is also called subject value scale and corresponds to the position of the subject along the measured variable continuum; *δ*_*i*_ is also called stimulus value scale and corresponds to the position of the stimulus along the same continuum of the measured variable. For example, in the case where the phenomenological-experimental task consists of determining the discriminative ability of a group of subjects, *β*_*n*_ represents the discriminative ability of the *n*th subject and *δ*_*i*_ the difficulty of the *i*th stimulus to be discriminated. Ability and difficulty can be represented along the same continuum in which the relationship of subject parameters and stimulus parameters are shown. As per Fig. 2 for example, the subject with ability *β*_4_ is able to discriminate difficult stimuli *δ*_3_ and *δ*_6_, but not *δ*_2_, *δ*_1_, *δ*_3_ and *δ*_4_.

The position of *β*_*n*_ and *δ*_*i*_ in Fig. 2 is related to the experimental data on the basis of the following logic:

1. if *β*_*n*_ falls to the right of *δ*_*i*_, it follows that the *n*th subject dominates the *i*th stimulus, or rather, *β*_*n*_ > *δ*_*i*_: the subject discriminates the stimulus;
2. if *β*_*n*_ falls to the left of *δ*_*i*_, it follows that the *i*th stimulus dominates the *n*th subject, or rather, *β*_*n*_ < *δ*_*i*_: the subject does not discriminate the stimulus;

It is relatively easy to pass from a linear transformation (the last equation shown) such as that of expression to the definition of a mathematical model that specifies the probability of obtaining a certain discriminative performance in relation to the scalar values *β*_*n*_ and *δ*_*i*_. To this end, the example of detection can be taken, or rather, the simplest discriminative performance: formally, the p probability of obtaining a detection (1 = yes detection; 0 = no detection) can be indicated as

$$ {\text{p}}\left\{ {{{\uplambda }}_{\text{ni}} = 1   {\text{or }}0|{{\upbeta }}_{\text{n}} ,{{\updelta }}_{\text{i}} } \right\} $$

Seeing as the numerical value of *λ*_*ni*_ falls within the range −∞ and +∞ and that of a probability value falls within 0 and 1, we can proceed towards the construction of a mathematical model, first reducing the range of variation between 0 and $+ \infty$ expressing *λ*_*ni*_ as an exponent of neperian e

$$ 0 < {\text{e}}^{{{{\uplambda }}_{\text{ni}} }} < + \infty $$

and then reducing the variation in terms of probability dividing by a normalization factor

$$ 0 < \frac{{{\text{e}}^{{{{\uplambda }}_{\text{ni}} }} }}{{1 + {\text{e}}^{{{{\uplambda }}_{\text{ni}} }} }} < 1 $$

Now we can express the probability of a yes-detection as follows

$$ {\text{p}}\left\{ {{{\uplambda }}_{\text{ni}} = 1|{{\upbeta }}_{\text{n}} ,{{\updelta }}_{\text{i}} } \right\} = \frac{{{\text{e}}^{{{{\uplambda }}_{\text{ni}} }} }}{{1 + {\text{e}}^{{{{\uplambda }}_{\text{ni}} }} }} $$

and probability of a no-detection

$$ {\text{p}}\left\{ {{{\uplambda }}_{\text{ni}} = 0|{{\upbeta }}_{\text{n}} ,{{\updelta }}_{\text{i}} } \right\} = \frac{1}{{1 + {\text{e}}^{{{{\uplambda }}_{\text{ni}} }} }} $$

The last two equations can represented together

$$ {\text{p}}\left\{ {{{\uplambda }}_{\text{ni}} = {\text{w}}_{\text{ni}} |{{\upbeta }}_{\text{n}} ,{{\updelta }}_{\text{i}} ,{\text{w}}_{\text{ni}} = \left( {0,1} \right)} \right\} = \frac{{{\text{e}}^{{{\text{w}}_{\text{ni }} {{\uplambda }}_{\text{ni}} }} }}{{1 + {\text{e}}^{{{\text{w}}_{\text{ni }} {{\uplambda }}_{\text{ni}} }} }} $$

Breaking down λ_ni_ into its scalar values, we obtain

$$ {\text{p}}\left\{ {{{\uplambda }}_{\text{ni}} = {\text{w}}_{\text{ni}} |{{\upbeta }}_{\text{n}} ,{{\updelta }}_{\text{i}} ,{\text{w}}_{\text{ni}} = \left( {0,1} \right)} \right\} = \frac{{{\text{e}}^{{{\text{w}}_{\text{ni}} \left( {{{\upbeta }}_{\text{n}} - {{\updelta }}_{\text{i}} } \right)}} }}{{1 + {\text{e}}^{{\left( {{{\upbeta }}_{\text{n}} - {{\updelta }}_{\text{i}} } \right)}} }} $$

**Fig. 3:**
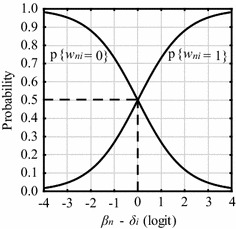
Simple logistic model: probability of a right/wrong response

This expression is the formalization of a Simple Logistic Model, known as Rasch Model (Rasch 1960; Andrich 1988), as is shown in Fig. 3.

Similar considerations can be made for the entire family of Rasch models, with both dichotomous and categorical data (Wright and Masters 1982).

### Mathematical demonstrations

It is possible to demonstrate that Rasch’s model has the three above-mentioned properties of fundamental measurement.

#### Linearity

Starting from the categorical response scale, raw scores gathered do not form interval measurement scales because of the non-linear metric typical of cumulative sequences in which a subject can obtain scores that oscillate from a minimum to a maximum, proceeding, step by step, by constant increments (Wright and Masters 1982). In cases as the one just described, the extreme categories of the ordinal scale of responses are flattened out. Raw scores show the phenomenon in question exactly the way a distorting mirror reflects an object; all the elements of the object appear to be laid out in a correct sequential way (think, for example, of a body: the head is above the chest and the chest is above the legs), but the proportions have been modified by a good deal (lengthened head, squeezed chest, distorted legs, etc.). By using a Rasch model it is possible to avoid this problem, by defining a particular unit of measurement, called logit, which has the same value all along the continuum of the latent variable. A logit is a mathematical unit which comes from the logarithmic form of the model and it arises from the relationship of probabilities that are mutually exclusive. The following shows a case of detection in which the probability of obtaining a yes detection (response 1, numerator) and the probability of not obtaining one (response 0, denominator) are related.

$$ logit = ln\left( {\frac{{\frac{{e^{{\left( {\beta_{n} - \delta_{i} } \right)}} }}{{1 + e^{{\left( {\beta_{n} - \delta_{i} } \right)}} }}}}{{\frac{1}{{1 + e^{{\left( {\beta_{n} - \delta_{i} } \right)}} }}}}} \right) = ln\left[ {e^{{\left( {\beta_{n} - \delta_{i} } \right)}} } \right] = \beta_{n} - \delta_{i} $$

Since it is the difference *β*_*n*_ − *δ*_*i*_ that governs the response probability, it is possible to add or subtract a constant of some type for different levels of ability of the subjects and of difficulty, in a general sense, of the stimuli without changing the relationship *β*_*n*_ − *δ*_*i*_. Therefore, the model is additive, something that is characteristic of a constant interval scale.

#### Specific objectivity

Starting from the performance, the relationship between stimulus parameters is not influenced by subject parameters. With two stimuli i and j and two subjects n and m, we can write

$$ \left( {\beta_{n} - \delta_{i} } \right) - \left( {\beta_{n} - \delta_{j} } \right) = \left( {\beta_{m} - \delta_{i} } \right) - \left( {\beta_{m} - \delta_{j} } \right) \Rightarrow \delta_{j} - \delta_{i} = \delta_{j} - \delta_{i} $$

that is, the relationship between the parameters of the two stimuli *i* and *j* are absolutely not influenced by the parameters *n* and *m* of the subjects (indeed, *β* disappears from the formula). The same can be said for the relationship between the subjects, which is not influenced by the stimuli (indeed, *δ* disappears from the formula)

$$ \left( {\beta_{n} - \delta_{i} } \right) - \left( {\beta_{m} - \delta_{i} } \right) = \left( {\beta_{n} - \delta_{j} } \right) - \left( {\beta_{m} - \delta_{j} } \right) \Rightarrow \beta_{n} - \beta_{m} = \beta_{n} - \beta_{m} $$

#### Stochastic independence

The probability associated with a pattern of responses given to *i* stimuli by a subject *n* is the same product of the response probabilities given to each of the *i* stimuli. Or rather

$$ p\left( {\lambda_{n1} ,\lambda_{n2} ,\lambda_{n3} , \ldots ,\lambda_{ni} } \right) = p\left( {\lambda_{n1} } \right) \cdot p\left( {\lambda_{n2} } \right) \cdot p\left( {\lambda_{n3} } \right) \cdot \cdots \cdot p\left( {\lambda_{ni} } \right) $$

This property is also called “local independence”. If the responses *λ*_*ni*_ and *λ*_*nj*_ of the same person to the two distinct stimuli *i* and *j* are considered, we can write

$$ p\left( {\lambda_{ni} ,\lambda_{nj} } \right) = p\left( {\lambda_{ni} } \right) \cdot p\left( {\lambda_{nj} } \right) $$

and presuming that the response pattern given is equal to (1,0), or (0,1), it follows that

$$ \begin{aligned} & p\left( {\lambda_{ni} = 1,\lambda_{nj} = 0} \right) \Rightarrow \left[ {\frac{{e^{{\left( {\beta_{n} - \delta_{i} } \right)}} }}{{1 + e^{{\left( {\beta_{n} - \delta_{i} } \right)}} }}} \right]\left[ {\frac{1}{{1 + e^{{\left( {\beta_{n} - \delta_{j} } \right)}} }}} \right] \\ & p\left( {\lambda_{ni} = 0,\lambda_{nj} = 1} \right) \Rightarrow \left[ {\frac{1}{{1 + e^{{\left( {\beta_{n} - \delta_{i} } \right)}} }}} \right]\left[ {\frac{{e^{{\left( {\beta_{n} - \delta_{j} } \right)}} }}{{1 + e^{{\left( {\beta_{n} - \delta_{j} } \right)}} }}} \right] \\ \end{aligned} $$

Considering that now the total scores of both patterns (1,0) and (0,1) are always equal to *t*_*n*_ = 1, the probability to be obtained *t*_*n*_ (that is, total score of subject *n*) can be written

$$ p\left( {t_{n} = 1} \right) = p\left( {\lambda_{ni} = 1,\lambda_{nj} = 0} \right) + \left( {\lambda_{ni} = 0,\lambda_{nj} = 1} \right) $$

It is now possible to define the conditional probability of a correct response to one of the two stimuli under consideration, posited as a condition of the total score *t*_*n*_ = 1. For *λ*_*ni*_ = 1

$$ \frac{{p\left( {\lambda_{ni} = 1,\lambda_{nj} = 0} \right)}}{{p\left( {t_{n} = 1} \right)}} = \frac{{\left[ {\frac{{e^{{\left( {\beta_{n} - \delta_{i} } \right)}} }}{{1 + e^{{\left( {\beta_{n} - \delta_{i} } \right)}} }}} \right]\left[ {\frac{1}{{1 + e^{{\left( {\beta_{n} - \delta_{j} } \right)}} }}} \right]}}{{\left[ {\frac{{e^{{\left( {\beta_{n} - \delta_{i} } \right)}} }}{{1 + e^{{\left( {\beta_{n} - \delta_{i} } \right)}} }}} \right]\left[ {\frac{1}{{1 + e^{{\left( {\beta_{n} - \delta_{j} } \right)}} }}} \right] + \left[ {\frac{1}{{1 + e^{{\left( {\beta_{n} - \delta_{i} } \right)}} }}} \right]\left[ {\frac{{e^{{\left( {\beta_{n} - \delta_{j} } \right)}} }}{{1 + e^{{\left( {\beta_{n} - \delta_{j} } \right)}} }}} \right]}} = \frac{{e^{{ - \delta_{i} }} }}{{e^{{ - \delta_{i} }} + e^{{ - \delta_{j} }} }} $$

Similarly for *λ*_*nj*_ = 1

$$ \frac{{p\left( {\lambda_{ni} = 0,\lambda_{nj} = 1} \right)}}{{p\left( {t_{n} = 1} \right)}} = \frac{{e^{{ - \delta_{j} }} }}{{e^{{ - \delta_{j} }} + e^{{ - \delta_{i} }} }} $$

Stochastic independence and conditional probability permit us to state that the probability of obtaining a precise response pattern for a generic subject *n* and stimuli *i*, given a value (in terms of total score) *t*_*n*_, is not related to parameter *β* of the subject, but it depends solely on parameter *δ* of the stimuli (this can be affirmed by the fact that parameter *β* disappears completely in the two previous formulas). Thus, we can speak of a sample-free condition. Similarly, it can demonstrated that the relative pattern of a generic stimulus *i* to which two subjects respond is not linked to the parameter *δ* of the stimulus, but solely to parameter *β* of the subjects (test-free condition). For *λ*_*ni*_ = 1, the probability of obtaining *s*_*i*_ (that is, the total score of stimulus *i*) is

$$ \frac{{p\left( {\lambda_{ni} = 1,\lambda_{mi} = 0} \right)}}{{p\left( {s_{i} = 1} \right)}} = \frac{{\left[ {\frac{{e^{{\left( {\beta_{n} - \delta_{i} } \right)}} }}{{1 + e^{{\left( {\beta_{n} - \delta_{i} } \right)}} }}} \right]\left[ {\frac{1}{{1 + e^{{\left( {\beta_{m} - \delta_{i} } \right)}} }}} \right]}}{{\left[ {\frac{{e^{{\left( {\beta_{n} - \delta_{i} } \right)}} }}{{1 + e^{{\left( {\beta_{n} - \delta_{i} } \right)}} }}} \right]\left[ {\frac{1}{{1 + e^{{\left( {\beta_{m} - \delta_{i} } \right)}} }}} \right] + \left[ {\frac{1}{{1 + e^{{\left( {\beta_{n} - \delta_{i} } \right)}} }}} \right]\left[ {\frac{{e^{{\left( {\beta_{m} - \delta_{i} } \right)}} }}{{1 + e^{{\left( {\beta_{m} - \delta_{i} } \right)}} }}} \right]}} = \frac{{e^{{ - \beta_{n} }} }}{{e^{{ - \beta_{n} }} + e^{{ - \beta_{m} }} }} $$

Similarly for λ_*mi*_ = 1

$$ \frac{{p(\lambda_{ni} = 0,\lambda_{mi} = 1)}}{{p(s_{i} = 1)}} = \frac{{e^{{ - \beta_{m} }} }}{{e^{{ - \beta_{m} }} + e^{{ - \beta_{n} }} }} $$

Therefore, it is necessary to emphasize that a response pattern does not have more information than that of the synthetic parameters *t*_*n*_ and *s*_*i*_. For this reason *t*_*n*_ and *s*_*i*_ are defined as “sufficient statistics”. All estimation procedures for the parameters *β*_*n*_ e *δ*_*i*_ have sufficient statistics for subjects and stimuli as a starting point (Baker 1987; Gustafsson 1980; Wright 1980; Andersen 1982; Rost 1982; Van den Wollenberg 1982; Molenaar 1983; Niemöller and Van Schuur 1983). Given these properties and the independence of the estimation of *β*_*n*_ with respect to *δ*_*i*_, it can be said that with regards to *δ*_*i*_, it is not necessary to hypothesize that the distribution of subjects is normal or otherwise: any type of distribution is acceptable (Andrich 1989). The same can be said for *β*_*n*_: it is not necessary to hypothesize any type distribution of the stimuli. With the model established, that is, the existing relationship between probabilities of emission of a certain response and scalar values *β*_*n*_ and *δ*_*i*_, it is important to adopt algorithmic procedures (Wright and Masters 1982) that start with experimentally gathered data (a simple two way table “stimulus X participants”) which lead to an estimation of the parameters *β*_*n*_ e *δ*_*i*_ on logit scales. Some of the most well-known estimation procedures are: JMLE (Joint Maximum Likelihood Estimation, Wright and Panchapakesan 1969), XMLE (eXclusory Maximum Likelihood Estimation, Linacre 1989), PROX (normal apPROXimation algorithm, Cohen 1979), MMLE (Marginal Maximum Likelihood Estimation, Bock and Aitkin 1981), CMLE (Conditional Maximum Likelihood Estimation, Follmann 1988), PMLE (Pairwise Maximum Likelihood Estimation, Rasch 1960; Choppin 1968, 1976, 1978, 1981), WMLE (Weighted Maximum Likelihood Estimation, Warm 1989).

## Discussion

### A Psychophysics application

The possibility of obtaining effective measurements on the ability of subjects (*β*_*n*_) and the difficulty of stimuli (*δ*_*i*_), are usually the point of arrival of psychophysical procedures by which experimental data are gathered. Psychophysics (Fechner 1860; Gescheider 1997; Guilford 1954; Kingdom and Prins 2016) has always been dedicated to revealing the intensity of mental processes linked to perception of physical phenomena capable of eliciting a sensation according to a model in which the intensity of the physical event is generally recorded directly through the use of measuring instruments capable of making quantifications that are considered objective (Campbell 1920) and the entity of perceptive phenomena is generally inferred according to Experimental Phenomenology procedures, starting from responses given from a sample of experimental subjects.

In these terms, a psychophysicist studies the laws of connections with physical measurements and judgements of a phenomenological character generated by the perception of the physical environment. It follows that psychophysical methods must therefore bring about identification of functional relationships between an objective continuum of physical stimuli *Φ* and a latent continuum of phenomenal judgements *Ψ*.

Situations exist, however, in which the classical psychophysical model cannot be used, in as much as the physical continuum *Φ* is not really available. Take, for example, the contrary dimensions of hot and cold: these dimensions exist only on a phenomenal level, that is, there is no unit of specific physical measurement to distinguish the two and to allow for quantification. In fact, a physicist would speak of a stimulus *Φ*_1_ that, for example, has a temperature of 500 K, or of a stimulus *Φ*_2_ with a temperature of 2000 K, but Kelvin degrees are units of measurements and they do not describe the sensation of hot or cold. Normally, a unit of physical measurement implies a sense of a specific dimension to the point that speaking of that unit puts ideas of what is being spoken about in that dimension into order and not that of others: if we say simply degrees Kelvin, we immediately understand that we are speaking of a temperature, but we do not understand precisely if it is hot or cold. This goes without saying in that, by definition, units of measurement are those parts of a continuum for which value 1 has been conventionally attributed. Therefore, if a unit of measurement is a portion of value 1 on continuum A, it cannot a portion of value 1 on continuum B at the same time, except where A and B are the same continuum. It follows that on the one hand temperature and hot–cold on the other cannot have the same unit of measurement in that the term temperature refers to a physical continuum, while the terms hot–cold refer to phenomenal continuums, and physical and phenomenal are certainly not the same thing. Thus, it goes without saying that degrees Kelvin is a measurement of temperature only, in as much as it is a portion of value 1 that is only on the physical continuum of temperature. If we were to try to carry out a classical psychophysical analysis of hot/cold by starting with degrees Kelvin, this would lead to an undesirable psychophysical function in reality that relates units of temperature measurements to phenomenal units.

Practically all psychophysical research conducted nowadays is based on the concept of Stevens’ defined measurement (1946), according to which “measuring is assigning numbers to objects based on rules.” According to Michell (1999), this associated definition together with ignoring that a construct, in order to be measurable, must have an additive structure, raises the doubt that both Experimental Psychology in general and Psychophysics in particular, ignore, to a great degree, the structures of the variables that they study. Dichotomous and ordinal data are often treated as interval scales and no verifying of the additive structure of the variables is carried out.

Scientific measurements require that measurements assigned to attributes/constructs are independent of the experimental subjects: this is not what happens in traditional Psychophysics where measurements are confused with people’s responses. Quantifying a stimulus from this viewpoint means admitting that the stimulus changes in relationship to the people to whom it is proposed. This is a big limit: to make a comparison with Physics, it is as if we said that 1000 K of the temperature of an object depends on who is measuring the temperature and not on the object itself. Traditional psychophysical research confuses calibration of stimuli with the measurement of the constructs (Wright and Stone 1979). Moreover, there are many researchers in the field of Human Sciences who entirely reject the idea of trying to measure psychological constructs because they are considered to be too complex to be subject to an “insulting process of reductionism to mere numbers” (Bond and Fox 2015). This bias seems to be generated both from the overrated opinion that the psychological-scientific community has of measurements made in physics and the underrated opinion it has of psychometric quantification procedures.

Nevertheless, and despite the fact that various alarm bells have been ringing throughout the history of experimental psychology (Loevinger 1947; Gulliksen 1950; Tucker 1953; Angoff 1960) the traditional mindset (Stevens) remains predominant, causing what Michell (1997) defines “confused methodological thinking”.

### A solution

A possible solution can be to describe the laws of connections between the physical and the phenomenal, starting with the scalar values *Ψ* of subjects (*β*_*n*_) and the scalar values *Φ* of stimuli (*δ*_*i*_) as above described.

With respect to the classical approach, this procedure allows for a quantification that measures characteristics of both stimuli and subjects (scalar values *δ*_*i*_ and *β*_*n*_), and it allows for the construction of a psychophysical function even if the physical continuum is not actually available (as in the case mentioned of the hot–cold contraries), in that the common psychophysical function *Ψ* = *f*(*Φ*) becomes the function *Ψ*_*i*_ = *f*(*δ*_*i*_), when for each scalar values *δi* exists a direct evaluation of the psychological sensation *Ψ*_*i*_. In addition, this procedure allows for the relationship *Ψ*_*n*_ = *g*(*β*_*n*_) between responses of subjects and their abilities to be studied, when for each scalar values *β*_*n*_ exists a direct subject’s evaluation *Ψ*_*n*_.

The classical psychophysical function *Ψ* = *f*(*Φ*) cannot be studied without stimuli *Φ*, but the functions *Ψ*_*i*_ = *f*(*δ*_*i*_) and *Ψ*_*n*_ = *g*(*β*_*n*_) can be used without any problems: stimuli *Φ* are only those things that produce the sensation, but we are not interested in their physical properties. Come to think of it, this is exactly what happens in the cases of phenomenological explanations: take, for example, the phenomenon of phenomenal transparency from Fuchs (1923) and Metelli (1974) in which they manipulate the stimuli, but the perceptive fact, namely transparency, is explained only by way of reference to other perceptive facts. Scalar values of stimuli, *δ*_*i*_, only come from responses given by subjects to the stimuli, from a quantification, which we could say is phenomenal, of the sensations, but they are certainly not the stimuli.

### A simulated study

In this context it seemed appropriate to refer to an simulated study of two-dimensional constructs that are typically phenomenal, the opposite qualities of hot and cold, in order to continue the above discussion.

*Stimuli* 20 objects Φ of the same shape and size which are different only for their surface temperature. Between a stimulus and the precedent and successive it is believed that constant temperature variation higher than the differential threshold is obtained.

*Participants* 100 subjects, evenly distributed by sex and age.

*Procedure* subjects touch (for a fixed time of t seconds) the surface of the objects with the palm of their hands, one at a time and at time intervals that have been studied so that there is no reciprocal influence. There are two experimental phases: in the first one, the subjects are asked to evaluate on a scale of 7 points (from 0 = “not at all” to 6 = “extremely”) how hot the object appears. At the end of the first phase, the second one begins where the participants are asked to evaluate on the same 7-point scale how cold an object appears. At the beginning of the experiment, the subjects touch the whole range of stimuli to get them used to the responses of the set of stimuli.

*Results* at the end of the experiment, two distinct data matrices (see structure of Table 1) are produced, with stimuli and subjects, one for each experimental phase (thus, one for hot and one for cold). The next step would be to carry out analysis of the data with the Extended Logistic Models, from the Rasch family of models (Andrich 1978a, b, c, 1988) which leads to an estimation of the scalar values *βn* and *δi* starting from the categorical data. There are many statistical softwares able to estimate the scalar values *βn* and *δi* starting from matrices as Table 1. A great open-source solution is eRm package (Mair and Hatzinger 2007) of the R-software environment for statistical computing and graphics (R Core Team 2016).

**Table 1:** Theoretical dataset: stimuli × subjects

	Stimuli *Φ*_*i*_					Average (subject) values
	*Φ*_1_	*Φ*_2_	*Φ*_3_	…	*Φ*_20_	
*Subjects S*_*n*_						
*S*_1_	*Ψ*_1-1_	*Ψ*_1-2_	*Ψ*_1-3_	*…*	*Ψ*_1-20_	*Ψ*_*S*1_
*S*_2_	*Ψ*_2-1_	*Ψ*_2-2_	*Ψ*_2-3_	*…*	*Ψ*_2-20_	*Ψ*_*S*2_
*S*_3_	*Ψ*_3-1_	*Ψ*_3-2_	*Ψ*_3-3_	*…*	*Ψ*_3-20_	*Ψ*_*S*3_
*…*	*…*	*…*	*…*	*…*	*…*	*…*
*S*_100_	*Ψ*_100-1_	*Ψ*_100-2_	*Ψ*_100-3_	*…*	*Ψ*_100-20_	*Ψ*_*S*100_
Average (stimulus) values	*Ψ*_*Φ*1_	*Ψ*_*Φ*2_	*Ψ*_*Φ*3_	*…*	*Ψ*_*Φ*20_	

Already other papers show how it is possible to use the Rasch models in Psychophysics. (Vidotto et al. 1996; Burro et al. 2011; Bianchi et al. 2011).

The scalar values *βn* and *δi* can be laid out along the same continuum (in units of logits), one for the hot variable and one for the cold variable, on which it is possible to compare stimuli and subjects according to the logic proposed in Fig. 2.

**Fig. 4:**
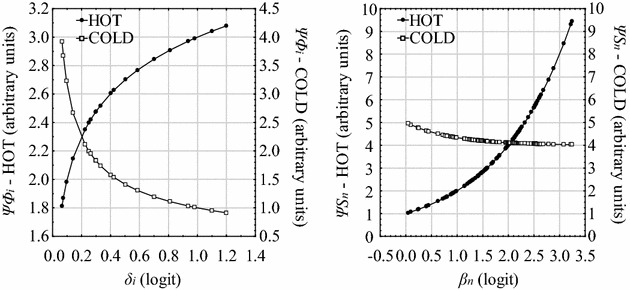
Distinct theoretical representation of possible functions *Ψ*_*i*_ = *f*(*δ*_*i*_) (on the *left*) and *Ψ*_*n*_ = *g*(*β*_*n*_) (on the *right*) of the properties hot and cold

In reference to the Table 1, the functions *Ψ*_*i*_ = *f*(*δ*_*i*_) and *Ψ*_*n*_ = *g*(*β*_*n*_) became respectively *Ψ*_*Φi*_ = *f*(*δ*_*i*_) and *Ψ*_*Sn*_ = *g*(*β*_*n*_). They can be represented as in Fig. 4.

## Conclusions

According to Wright and Stone (1999), no scientific method is complete if it is not based on a concept of fundamental measurement.

What follows are some clarifications on the above explained procedure and on its position in the scientific research. It doesn’t mean that in order to make discoveries or to give scientific explanations it is necessary to have an authorized method that respects the principles of fundamental measurement. The cited properties of linearity, stochastic independence and specific objectivity have always been associated with the concept of measurement, and not that of discovery and explanation. Certainly, a good mathematical model can produce qualitatively better and more precise measurements. Therefore, the idea is that a more refined instrument can, in certain situations, provide a better description and explanation of an already known phenomenon, while in other situations it may lead to a discovery. The example of the telescope seems fitting: in some cases, a telescope with a better power of resolution allows one to see an already known planet better, but in other cases it may lead to the identification of new ones, that is, discoveries.

In the same way, the proposal procedure as was seen above sometimes describes the correct relationship between the world, subject and experience, and in other cases it means using an approach for the study of new facts.

There are few psychologists and sociologists who are aware of this. This is true not so much because they disapprove of fundamental measurement, but only because for the most part they do not know how to obtain it. In the pages of this paper the attempt has tried to make up for this lack at least partially, by showing that it is possible to carry out a fundamental measurement starting from Experimental Phenomenology by way of the Theory of Conjoint Measurement (Luce and Tukey 1964).

The Theory of Conjoint Measurement recognizes that the idea of measuring, as it is meant in Physics, is too simple to be used in psychosocial research. Physics has to do with objects that allow for operations of concatenation: it is easy to show that the weight of two bags together is the same as the sum of the single sacks weighed individually.

Proposing, as it has been done here, the Rasch model as a point of contact between Experimental Phenomenology, and the Theory of Conjoint Measurement does not mean that it is not possible to use other mathematical transformations which allow for the carrying out of the same considerations. In fact, the Theory of Conjoint Measurement does not specify what the monotone function has to be that links the probability that a subject gives one particular response on the one hand, and the ability of the subject and difficulty of the stimulus on the other hand. Speaking of this, in the psychometric literature (Lord and Novick 1968), two well-known functions are usually considered: normal inverse and logistic inverse. Both of these have properties of fundamental measurement, but the latter (which is none other than a Rasch model) offers better statistical properties for the estimation of ability (β_n_) and difficulty (*δ*_*i*_) parameters, starting from the raw data received in an experimental task.

Concerning types of cumulative stochastic models, the Rasch family of models focuses on the evaluation of two expectations (Rasch 1960; Wright and Stone 1979). Taking the case of detection, for example a subject having better discriminative ability must be associated with a greater response emission probability of a yes detection to any of the stimuli under examination with respect to a less capable subject, and it is more probable that a subject has a detection with an easy stimulus than with a difficult one.

Given that, by definition, a measurement according to Rasch’s analysis is not deterministic, deviations from expectations specified above in the data during the experiment can be recorded. And, it is possible that certain subjects respond that they perceived difficult stimuli and did not perceive the easy ones. In other words, it may happen that subjects may display behaviour that is non-cumulative/non-unidimensional.

Quantification and successive evaluation of discrepancies that exist among response matrices and expectations emerging from a Rasch analysis are indicators to the experimenter of the quality of the measurements obtained (Duncan 1984).
